# Roles of protein crotonylation in tumors: regulatory mechanisms and therapeutic potential

**DOI:** 10.3389/fimmu.2026.1952793

**Published:** 2026-10-23

**Authors:** Yaqian Liang, Qinyue Liu, Can Yang, Yi Xun, Yanglin Liu, Yingying Shen, Xuyu Zu

**Affiliations:** 1 Cancer Research Institute, The First Affiliated Hospital, Hengyang Medical School, University of South China, Hengyang, Hunan, China; 2 Hunan Provincial Clinical Medical Research Center for Drug Evaluation of Major Chronic Diseases, The First Affiliated Hospital, Hengyang Medical School, University of South China, Hengyang, Hunan, China; 3 Department of Clinical Laboratory Medicine, Institution of Microbiology and Infectious Diseases, Hunan Province Clinical Research Center for Accurate Diagnosis and Treatment of High-incidence Sexually Transmitted Diseases, The First Affiliated Hospital, Hengyang Medical School, University of South China, Hengyang, Hunan, China

**Keywords:** lysine crotonylation, protein post-translational modification, the immune microenvironment, therapeutic strategy, tumor metabolic reprogramming

## Abstract

Lysine crotonylation(Kcr), an important protein post-translational modification, establishes a connection between cellular metabolism and epigenetic regulation. This modification utilizes crotonyl-CoA as a donor and is co-regulated by “writers”, “erasers” and “readers”. Furthermore, it participates in key biological processes including gene transcription, metabolic reprogramming, DNA damage repair, and remodeling of the tumor immune microenvironment. Recent studies have demonstrated that crotonylation is dysregulated in various malignant tumors, including breast cancer, colorectal cancer, liver cancer, lung cancer, and glioma. It drives tumor proliferation, invasion, metastasis, and drug resistance by influencing metabolic enzyme activity, chromatin status, DNA repair efficiency, and immune signaling pathways. Lysine crotonylation demonstrates significant environmental-dependence across different cancers; Not only can it promote the malignant phenotype of tumor cells, but it can also potentiate the response to immunotherapy under specific metabolic conditions. Preclinical studies have explored therapeutic strategies targeting tumor crotonylation, including metabolic interventions, enzymatic regulation, and combination immunotherapy; however, these approaches remain investigational and have not been clinically validated. This narrative review summarizes the molecular mechanisms, biological functions, and current advances of crotonylation in diverse malignancies, and highlights unresolved questions and emerging therapeutic hypotheses.

## 1 Introduction

Protein post-translational modifications (PTMs) are a fundamental molecular mechanism regulating gene expression, protein function, and cellular phenotypes. Among various PTMs, lysine acylation modifications have emerged as a prominent focus in cancer research (1). As a newly identified acylation modification, lysine crotonylation is characterized by unique chemical structure and regulatory patterns that distinguish it from canonical acetylation. It dynamically modulates intracellular crotonyl-CoA pools to translate metabolic signals into epigenetic regulatory responses. Since its systematic identification as a novel histone mark in 2011 (2), research on crotonylation has broadened significantly—from initial studies centered on histones to the discovery of diverse non-histone substrates (3, 4). Its regulatory network comprises the canonical triad of “writers”, “erasers” and “readers” and it actively participates in multiple critical biological processes, including transcriptional activation (5), metabolic reprogramming (6), DNA damage repair (7), cell cycle regulation (8), and modulation of the immune microenvironment (9).In the initiation and progression of tumors, metabolic reprogramming and epigenetic dysregulation are highly coupled, jointly driving malignant transformation (10), invasion and metastasis (11), and therapy resistance (12). A multitude of studies suggest that diverse tumor cells display abnormalities in lysine catabolism and fatty acid metabolism, resulting in the reshaping of intracellular crotonyl - CoA levels and the subsequent global imbalance of crotonylation modification (12). Crotonylation can impact the transcription of oncogenes (10) or tumor suppressor genes (13), by modulating histone modification status, or directly modify non - histone substrates such as metabolic enzymes (14), DNA repair proteins (7), and signaling molecules (15), thereby altering protein stability, enzymatic catalytic activity, and protein - protein interaction networks, and regulating key pathways such as glycolysis and lipid synthesis (16–18). Simultaneously, crotonylation is involved in the regulation of immune signals such as cGAS-STING and type I interferon, influencing CD8+ T cell infiltration, tumor-associated macrophage polarization, and the efficacy of immune checkpoint inhibitors, thus emerging as a crucial hub connecting tumor metabolism and immune evasion (19). At present, the aberrant expression and functional mechanisms of crotonylation are gradually being uncovered in various malignant tumors, including breast cancer (9), colorectal cancer (13), liver cancer (20), pancreatic cancer (17), lung cancer (21), glioma (19)and cervical cancer (7), yet its mode of action shows cancer - type heterogeneity. Some site-specific modifications facilitate tumor progression (22), while others exhibit tumor-suppressive activities (9). With advances in crotonyl-proteomics and site-specific functional studies, intervention strategies targeting lysine crotonylation, including metabolic modulation, small-molecule inhibitors against crotonylation “writers” and “erasers, “ and combination immunotherapies, are being explored in preclinical models (9, 12, 23). However, current evidence remains predominantly preclinical and context-specific, and the clinical translation of these strategies requires further validation. This narrative review summarizes the molecular definition, regulatory mechanisms, and core biological functions of lysine crotonylation, emphasizes its context-dependent roles and key signaling axes across tumor types, and outlines crotonylation-based therapeutic hypotheses and associated knowledge gaps. Collectively, this review aims to provide a conceptual framework for understanding the interplay between metabolism and epigenetic regulation in tumorigenesis and to highlight candidate mechanisms for future investigation, rather than to define clinically actionable targets.

## 2 Crotonylation

### 2.1 Definition of crotonylation

Lysine crotonylation is a metabolism-associated epigenetic modification that has attracted significant research attention in recent years. It represents a reversible post-translational modification in which a crotonyl group (CH_3_–CH=CH–CO–) is covalently attached to the ϵ-amino group of lysine residues. This process follows the canonical “writer–eraser–reader” regulatory model. In 2011, Tan et al. were the first to systematically identify lysine crotonylation as a novel histone mark, thereby establishing the conceptual and experimental foundation for subsequent investigations into this modification (2). Subsequent studies demonstrate that lysine crotonylation occurs not only on histones but also extensively occurs on non-histone proteins—including metabolic enzymes, DNA repair factors, and signaling molecules—underscoring its cross-pathway regulatory roles (3, 4). Crotonyl-CoA acts as the direct donor for lysine crotonylation, and its intracellular concentration is co-regulated by fatty acid oxidation, lysine catabolism, and the cellular conversion of exogenous crotonate. Key enzymes (including ACSS2, GCDH, ECHS1, CDYL, and ACOX2) modulate the pool of crotonyl-CoA available for lysine crotonylation by regulating its biosynthesis and consumption (6, 24–27). Notably, Sabari et al. demonstrated that fluctuations in intracellular crotonyl-CoA levels directly modulate p300-catalyzed histone crotonylation, thereby altering transcriptional output. Their findings definitively established a causal chain linking metabolite dynamics to epigenetic modification and, ultimately, to gene expression regulation (28). As a metabolism-driven epigenetic mark, lysine crotonylation—orchestrated through crotonyl-CoA homeostasis—plays a pivotal role in diverse fundamental biological processes, including transcriptional regulation (28, 29), DNA damage repair (30), metabolic reprogramming, and immune microenvironment modulation (25, 31), serving as a mechanistic bridge between cellular metabolism and epigenetics.

### 2.2 Regulatory mechanisms of crotonylation

The crotonylation process is dynamically regulated by a suite of dedicated enzymes—including writers and erasers—as well as reader proteins and the Crotonyl-CoA conjugate. This post-translational modification targets both histone and non-histone proteins (**Table 1**).

**Table 1:** The modulatory factors and substrate modification sites associated with histone and non-histone Kcr.

Category	Enzymes	Targets and sites		Functions	Ref.
		Histone	Nonhistone		
Writers	p300	H3K18	—	Regulate transcriptional;	(28)
		HNRNPA1	—	promotes tumor progression;	(32)
	CBP	—	ENO1-K420cr	Regulate enzymatic activity;	(14)
		—	PRKACA-K84cr	Regulate enzymatic activity;	(33)
	MOF (KAT8/hMOF)	H3K4cr, H3K9cr, H3K18cr, H3K23cr, H4K8cr, H4K12cr	—	Regulate transcription by chromatin remodeling;	(34)
	Gcn5/Esal	H3K9cr, H3K14cr, H3K18cr	—	Regulate transcription;	(35)
	GCN5	—	DNA-PKcs-K525cr	Regulate DNA damage response and repair	(36)
	TIP60	—	EB1-K66cr	Regulates mitosis and spindle localization;	(37)
	KAT7 (HBO1)	—	CANX-K525cr	Regulates mTORC1 activity;	(38)
	PCAF	—	NPM1	Regulates enzyme activity;	(39)
		—	Ku80-K568cr	Promotes NHEJ repair and radiotherapy resistance;	(22)
Erasers	HDAC1	H3K4cr, H3K9cr, H3K23cr, H4K8cr, H3K18cr, H4K12cr	—	Repress transcriptional;	(40)
	HDAC2/3	H3K4cr, H3K9cr, H4K8cr, H4K12cr, H3K18cr	—	Regulates transcription;	(40)
	HDAC6	—	Lamin A-K265cr/K270cr	Maintains subcellular position;	(23)
	HDAC8	H3K4cr, H3K9cr, H4K8cr, H4K12cr, H3K18cr —	Ku80-K568cr	Regulates transcription; Regulates DNA damage response and repair	(40) (22)
	SIRT1	H3K9cr, H4K8cr	—	Regulates transcription;	(40)
	SIRT2	—	ENO1-K420cr	Regulates enzyme activity;	(14)
		—	SEPT2-K74cr;	Regulates enzyme activity;	(20)
	SIRT3	—	PRKACA	Regulates enzyme activity;	(33)
		—	cGAS-K254cr	DNA damage response and repair;	(7)
	SIRT3	—	MCM6-K599cr	Regulate DNA damage repair;	(41)
Readers	AF9 (YEATS)	H3K9cr, H3K18cr	—	Regulates transcription;	(42)
	YEATS2	H3K27cr	—	Regulates transcription;	(43)
	Taf14	H3K9cr	—	Regulates transcription;	(44)
	MOZ/DPF2	H3K14cr		Regulates transcription;	(45)
Metabolic regulator	ACSS2	—	EZH2-K348cr	Provides crotonyl-CoA and regulate protein stability;	(9)
		H3K9cr	—	Provides crotonyl-CoA, the direct acyl donor for histone crotonylation;	(24)
	ECHS1	—	PGK1-K131cr	Depletion of crotonyl-CoA, regulates glycolysis and TCA cycle;	(6)
	GCDH	—	PGD-K163cr, TKT-K140cr, ALDOC-K28cr	Provides crotonyl-CoA, regulates glucose and immune microenvironment;	(46)
	ACADS	—	—	Maintains crotonyl-CoA metabolic homeostasis;	(27)
	ACOX2	H2b K86cr	Ehhadh (K329cr/K344cr/K572cr)	Regulate crotonyl-CoA metabolism;	(47)
	CDYL	—	RPA1(K88cr/K379cr/K595cr)	Regulates homologous recombination repair;	(26)
	CDYL1	H2BK12cr, H3K9cr, H3K27cr, H4K8cr, H3K18cr, H3K23cr, H3K79cr	—	Regulates transcription;	(48)

#### 2.2.1 Writers - crotonyltransferases

Histone crotonyltransferases (HCTs) catalyze the transfer of the crotonyl group to lysine residues on histone proteins. Given the structural similarity between crotonylation and acetylation, these two post-translational modifications share partial regulatory mechanisms. Accumulating evidence indicates that three major classes of histone acetyltransferases (HATs)—namely, p300/CBP, the MYST family and GCN5—also exhibit crotonyltransferase activity (49). In 2015, Sabari et al. demonstrated that p300/CBP was the primary histone crotonyltransferases (28). Subsequent studies further confirmed that p300 mediates lysine crotonylation and exerts stronger transcriptional activation than lysine acetylation at specific genomic loci (28). Liu et al. discovered that MOF exhibits crotonyltransferase activity in 2017 (34), but CBP/p300 remains the predominant crotonyltransferase in mammals. Members of the MYST protein family, including human MOF and its yeast homolog ESA1, catalyze histone lysine crotonylation at multiple sites (34), functioning as components of protein complexes *in vivo* (50). Kollenstart et al. demonstrated that Gcn5/Esa1 catalyzes site-specific lysine crotonylation and participates in transcriptional regulation in yeast (35). Specifically, GCN5 catalyzes histone H3 crotonylation at residues K9, K14, K18, K23, and K27 in Saccharomyces cerevisiae (35). Moreover, crotonyltransferase can target non-histone substrates. For instance, TIP60 catalyzes the crotonylation of EB1 at lysine 66 (K66), thereby regulating mitotic progression (37); lysine acetyltransferase 7 (KAT7) (also known as HBO1) mediates the crotonylation of calnexin (CANX) at K525 (38); and nucleophosmin 1 (NPM1), a non-histone protein, is crotonylated by p300/CBP-associated factor (PCAF) (39).

#### 2.2.2 Erasers - decrotonylases

Decrotonylases primarily catalyze the removal of Kcr from protein substrates, specifically histones. Bao et al. first demonstrated that silent information regulator proteins 1, 2, and 3 (SIRT1/2/3) exhibit decrotonylase activity, and revealed that SIRT3 acts as a key regulator of histone crotonylation and transcriptional programs (51). The histone deacetylase (HDAC) family has also been shown to exhibit decrotonylase activity. Wei et al. demonstrated that Class I HDACs (1, 2, 3, and 8) function as the principal histone decrotonylases in mammalian cells (40). Specifically, HDAC2 modulates the crotonylation levels of histone H2B at lysine12 (52); whereas HDAC3 reduces AKT1 crotonylation upon its own upregulation (53). In contrast, HDAC6 promotes the accumulation of crotonylation on lamin A at K265 and K270 (23). HDAC8 facilitates non-homologous end joining (NHEJ) repair and confers radiotherapy resistance by removing the crotonyl mark at Ku80 K568, thereby creating a permissive site for subsequent SUMOylation (22).Finally, SIRT7 catalyzes the decrotonylation of ribonucleotide reductase regulatory subunit M2 (RRM2)—a protein implicated in drug resistance—at K283 (15).

#### 2.2.3 Readers - crotonylation reader proteins

Crotonylation reader proteins mediate the specific recognition of crotonylated lysine residues and translate these post-translational modifications into downstream functional outcomes. To date, the major classes of structurally and functionally characterized crotonylation readers include the YEATS family, the DPF family, and select bromodomain-containing proteins (42, 54, 55). The YEATS family comprises Yaf9, ENL, AF9, Taf14, and Sas5 (54). AF9 was the first human crotonylation reader to be systematically characterized; its YEATS domain exhibits markedly higher binding affinity for H3K9cr and H3K18cr than for their acetylated counterparts, due to an open aromatic pocket that selectively engages the crotonyl moiety via π–π interactions (42). While structural studies indicate that YEATS family members share a conserved aromatic recognition mechanism, they display distinct site selectivity, as exemplified by YEATS2’s specific recognition of H3K27cr (43). Notably, the yeast Taf14 YEATS domain also specifically binds H3K9cr, underscoring the evolutionary conservation of this recognition mechanism (44). Collectively, these findings establish the YEATS family as a central and evolutionarily conserved module for lysine crotonylation recognition. The DPF family represents a second major class of crotonylation readers. The DPF domains of MOZ and DPF2 selectively bind H3K14cr, with specificity conferred by a hydrophobic terminal pocket within their PHD1 fingers that is precisely shaped to accommodate both the length and conformation of the crotonyl group (45).

#### 2.2.4 Crotonyl-CoA

In lysine crotonylation research, crotonyl-CoA serves as the central acyl donor that governs both the extent and specificity of this post-translational modification. As a short-chain acyl derivative of coenzyme A, crotonyl-CoA serves not only as the direct crotonyl group donor for lysine crotonylation but also as a critical metabolic intermediate in mitochondrial and peroxisomal pathways. Enzymes involved in the biosynthesis and catabolism of crotonyl-CoA—such as acetyl-CoA synthetase 2 (ACSS2) (24), short-chain enoyl-CoA hydratase 1 (ECHS1) (6), glutaryl-CoA dehydrogenase (GCDH) (46), short-chain acyl-CoA dehydrogenase (ACADS) (27), and acyl-CoA oxidases1and 2 (ACOX1–2) (27, 47)—thereby act as key regulators of lysine crotonylation. Chromodomain Y-like (CDYL) is a chromatin-associated transcriptional corepressor (56). Notably, CDYL also exhibits intrinsic crotonyl-CoA hydratase activity, enabling it to modulate histone crotonylation levels, and this enzymatic function is mechanistically linked to its role in transcriptional repression (26).

### 2.3 Biological functions of crotonylation

In recent years, accumulating evidence from extensive research has revealed that crotonylation plays a pivotal role in diverse pathophysiological processes. Beyond its canonical role in transcriptional regulation, crotonylation also contributes to cellular metabolic reprogramming, modulation of the tumor immune microenvironment, and regulation of the cellular response to DNA damage (**Figure 1**).

**Figure 1:**
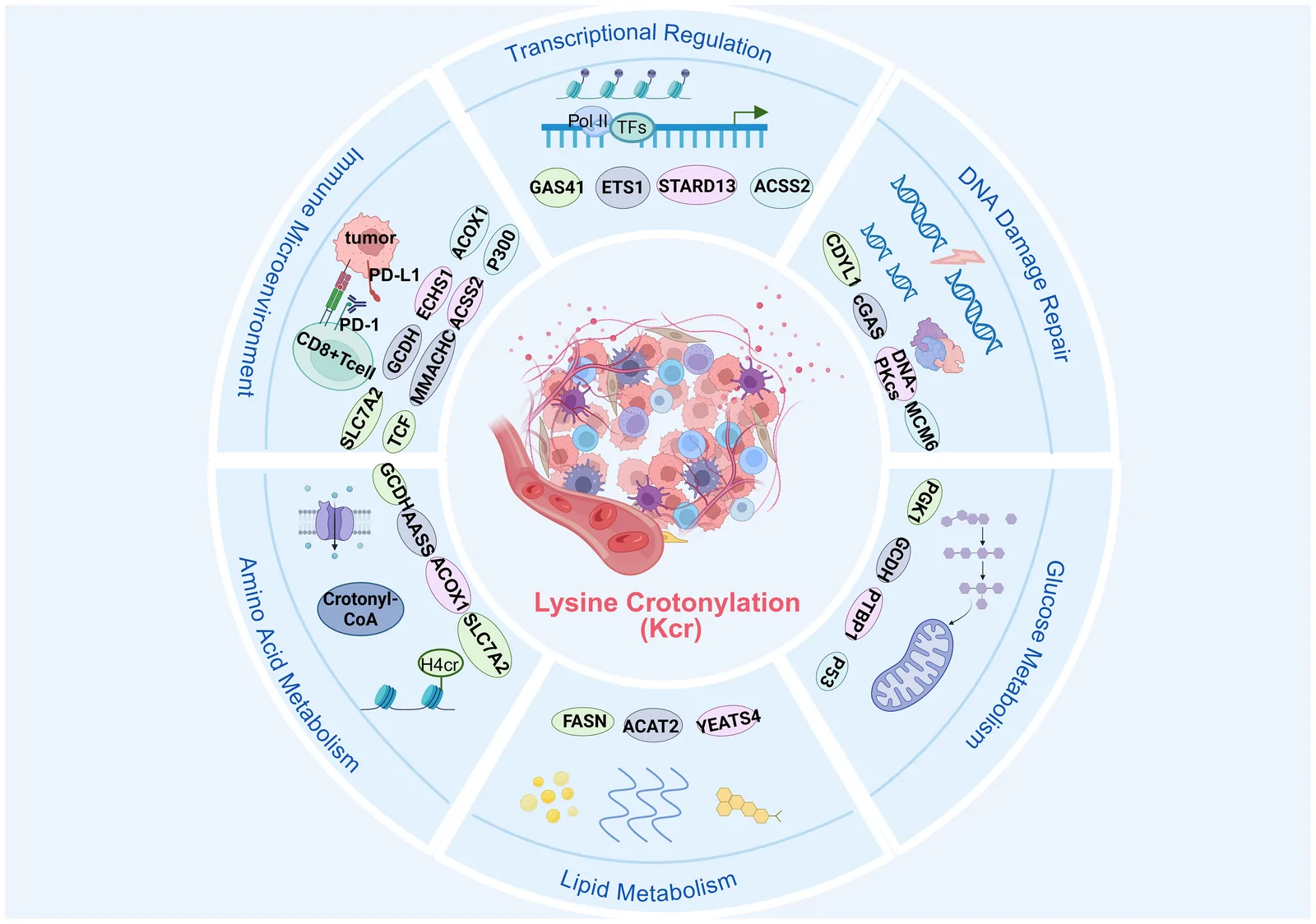
Proposed biological functions of Kcr in cancer. Kcr regulates six major cancer-related processes: transcriptional regulation, DNA damage repair, glucose metabolism, lipid metabolism, amino acid metabolism, and the immune microenvironment. Representative Kcr-related factors are shown in each sector. Arrows indicate promotion or activation.

#### 2.3.1 Transcriptional regulation

Throughout tumor initiation and progression, lysine crotonylation modulates tumor cell proliferation, metastasis, and drug resistance by remodeling chromatin architecture, recruiting transcriptional regulatory complexes, and reprogramming key gene expression networks. The YEATS domain-containing protein 4 (GAS41) recognizes both histone H3 lysine 27 acetylation (H3K27ac) and crotonylation (H3K27cr), and subsequently recruits the BRD2–MED14/MED23 transcriptional co-activator complex. This recruitment promotes the transcription of nuclear shape regulators—including LMNB1, LMNB2, SYNE4, and LEMD2—thereby maintaining nuclear morphology and sustaining the proliferative capacity of tumor cells (5). LINC00922 promotes tumor metastasis by increasing H3K27cr levels at the ETS1 (ETS proto-oncogene 1, transcription factor) promoter and enhancing ETS1 transcription through the sequestration of the decrotonylase SIRT3, thereby preventing its enzymatic activity at this locus (10). In contrast, the MMP14 (matrix metalloproteinase 14) transcript acts as a molecular scaffold, specifically binding SIRT3 and recruiting it to the STARD13 (DLC2) promoter. Local SIRT3-mediated decrotonylation reduces H3K27cr levels, induces chromatin compaction, and suppresses the transcription of the tumor suppressor STARD13, ultimately facilitating colorectal cancer metastasis (13). Furthermore, downregulation of ACSS2 diminishes intracellular crotonyl-CoA pools, resulting in reduced H3K56cr and consequent transcriptional repression of hepatocyte nuclear factor 1α (HNF1A). This repression leads to the aberrant activation of the PI3K/AKT signaling pathway, which consequently confers resistance to EGFR tyrosine kinase inhibitors (EGFR-TKIs) within tumor cells (12).

#### 2.3.2 DNA damage repair

Protein crotonylation has emerged as a pivotal post-translational modification regulating the DNA damage response (DDR) and repair pathways during tumorigenesis. Accumulating evidence indicates that crotonylation not only mediates transcriptional repression triggered by DNA double-strand breaks (DSBs) but also directly modulates DSB repair and the cellular response to replication stress. Upon DSB induction by the AsiSI endonuclease, CDYL1 suppresses H3K9cr through its crotonyl-CoA hydratase activity, leading to the dissociation of the transcriptional elongation factor ENL and resulting transcriptional inhibition (48). Ionizing radiation (IR)-induced genomic DNA damage modulates the crotonylation of cyclic GMP-AMP synthase (cGAS) at lysine 254. Under physiological conditions, this residue is constitutively crotonylated by CREB-binding protein (CBP); however, DNA damage triggers SIRT3-mediated decrotonylation at K254, which attenuates the DNA-binding affinity of cGAS and impairs its interaction with PARP1—thereby promoting homologous recombination (HR) repair in tumor cells (7). GCN5 catalyzes the crotonylation of the DNA-dependent protein kinase catalytic subunit (DNA-PKcs) at lysine 525, whereas HDAC3 mediates the decrotonylation; this dynamic, opposing regulation dictates DNA-PK complex assembly and non-homologous end joining (NHEJ) repair efficiency (36). Minichromosome maintenance protein 6 (MCM6), a core component of the DNA replication machinery, is essential for replication fork progression. Under replication stress, MCM6 K599 crotonylation is upregulated due to reduced SIRT7-mediated decrotonylation and RNF8-mediated ubiquitination. Elevated K599cr crotonylation impairs MCM2–7 complex assembly, thereby aggravating replication stress and DNA damage (41). SIRT7 acts as a decrotonylase that removes MCM6 K599 crotonylation and maintains its low basal level.

#### 2.3.3 Metabolic reprogramming

Tumor metabolic reprogramming refers to the coordinated, dynamic, and sustained reconfiguration of glucose, lipid, and amino acid metabolism in tumor cells, orchestrated by genetic and epigenetic mechanisms. This process supports malignant phenotypes such as rapid proliferation, invasion, and metastasis, as well as therapy resistance and survival under adverse microenvironmental conditions like hypoxia, nutrient scarcity, and extracellular acidosis. It represents a defining hallmark that distinguishes malignant cells from their normal counterparts.

##### 2.3.3.1 Glucose metabolism

Accumulating evidence indicates that lysine crotonylation is a pivotal post-translational modification that bridges tumor metabolic substrates with glucose metabolic reprogramming. Studies demonstrate that under hypoxic conditions, ECHS1 expression is upregulated and facilitates its physical interaction with phosphoglycerate kinase 1 (PGK1), leading to reduced lysine crotonylation of PGK1. This reduction in crotonylation activates PDHK1, suppresses pyruvate dehydrogenase (PDH) activity, restricts mitochondrial pyruvate entry, and consequently acetyl-CoA production, attenuates tricarboxylic acid (TCA) cycle efficiency, and concurrently enhances glycolytic flux in tumor cells, thereby driving hypoxia-induced metabolic reprogramming (6). GCDH elevates intracellular crotonyl-CoA levels, thereby augmenting global lysine crotonylation, which subsequently modulates key proteins involved in the pentose phosphate pathway and glycolysis, effectively suppressing the Warburg effect in tumor cells and inhibiting tumor progression (46). In colorectal cancer, KAT2B-mediated crotonylation of PTBP1 at K266cr enhances its association with hnRNPA1 and hnRNPA2, promoting the alternative splicing of PKM pre-mRNA toward the PKM2 isoform and increasing glycolytic flux (16). Collectively, lysine crotonylation orchestrates the reprogramming of glucose metabolism in tumor cells and modulates malignant progression by targeting critical metabolic enzymes, RNA splicing regulators and tumor suppressor proteins.

##### 2.3.3.2 Lipid metabolism

Crotonylation functions as a critical interface between epigenetic and metabolic processes, directing tumor lipid metabolic reprogramming by governing essential determinants of fatty acid synthesis and oxidation. Within neoplastic contexts, crotonylation reshapes fatty acid metabolism via a bimodal regulatory strategy that integrates “metabolic enzyme modification” and “chromatin reading.” In pancreatic ductal adenocarcinoma, the core lipogenic enzyme fatty acid synthase (FASN) is subject to site-specific crotonylation, primarily at lysine 235 (K235); this post-translational modification is dynamically controlled by the acetyltransferase p300 and the deacetylases HDAC1/3, thereby mechanistically linking intracellular crotonyl-CoA levels to lipogenic output (3). Another study in pancreatic cancer demonstrated that elevated expression of GCDH enhances CBP-mediated crotonylation of acetyl-CoA acetyltransferase 2 (ACAT2) at K235. This post-translational modification disrupts Insig1-dependent retention of the SCAP–SREBP2 complex in the endoplasmic reticulum, thereby enabling its trafficking to the Golgi apparatus and subsequent proteolytic activation—ultimately driving cholesterol biosynthesis (17). Moreover, the YEATS4 reader domain selectively binds histone H3 lysine 14 crotonylation (H3K14cr), driving transcriptional upregulation of CD36, CPT1A and ACOX1. This signaling cascade enhances fatty acid uptake and mitochondrial β-oxidation, thereby maintaining breast cancer stem cell–like traits and tumor growth (57).

##### 2.3.3.3 Amino acid metabolism

The interplay between amino acid metabolism and protein crotonylation is fundamentally regulated by the intracellular concentration of crotonyl-CoA (19). The saccharopine pathway serves as the principal route for lysine catabolism. In glioblastoma, augmented lysine uptake, driven by the lysine transporter SLC7A2, coupled with upregulated GCDH expression and diminished ECHS1 expression, culminates in intracellular crotonyl-CoA accumulation, thereby elevating histone H4 lysine crotonylation. This epigenetic modification suppresses the MDA5/cGAS–mediated type I interferon signaling pathway, thereby facilitating tumor immune evasion. Notably, dietary lysine restriction reverses this immunosuppressive cascade and exhibits synergistic effects with immune checkpoint blockade to inhibit tumor progression (19). Furthermore, SLC7A2 regulates lysine catabolism to drive CD8+ T cell activation. Specifically, it elevates the expression of acyl-CoA oxidase 1 (ACOX1), which in turn enhances histone crotonylation in CD8+ T cells and promotes the interaction between ACOX1 and TCF1, thereby strengthening anti-tumor T cell immunity (18).

#### 2.3.4 Immune microenvironment and immune response

As a novel post-translational modification, crotonylation assumes a central function in tumor biology, driving tumor metabolic reprogramming and governing gene expression, while simultaneously shaping the tumor immune microenvironment to regulate tumor–immune crosstalk.In glioblastoma, tumor stem cells orchestrate a reprogramming of lysine metabolism via the SLC7A2–GCDH–ECHS1 axis, culminating in the accumulation of crotonyl-CoA and heightened histone crotonylation. This epigenetic alteration represses the MDA5/cGAS–type I interferon signaling cascade, curtails CD8+ T cell infiltration, and consequently promotes immune evasion. Targeted pharmacological disruption of this metabolic–epigenetic axis synergizes with anti-PD-1 immunotherapy to elicit robust antitumor efficacy, highlighting histone crotonylation as a critical molecular nexus that bridges amino acid metabolism with tumor-mediated immune suppression (19). In breast cancer, the SLC7A2–ACOX1–TCF1 signaling axis selectively augments histone crotonylation within CD8+ T cells, thereby driving their functional activation and potentiating their responsiveness to anti-PD-1 therapy, which collectively reinforces antitumor immunity (18). Another study revealed that crotonate activates the ACSS2–p300 signaling pathway, ACSS2 provides crotonyl-CoA, indirectly regulates EZH2-K348cr. This post-translational modification promotes EZH2 ubiquitination and subsequent proteasomal degradation, which lowers global H3K27me3 levels and reverses the immunosuppressive tumor microenvironment. As a result, tumor metastasis is suppressed, and the therapeutic efficacy of anti-PD-L1 blockade is markedly augmented, underscoring the considerable clinical translational promise of crotonylation-targeted combination strategies (9). In a retrospective analysis of HCC patient cohorts, low GCDH expression was associated with elevated infiltration of PD-1+ CD8+ T cells and with enhanced responsiveness to anti-PD-1 therapy, suggesting that crotonylation-associated biomarkers may aid immune stratification (46). In lung adenocarcinoma, investigations into histone crotonylation have largely focused on its translational utility as a biomarker: the crotonylation-linked gene MMACHC demonstrates associations with immune cell infiltration, patient prognosis, and immunotherapy response. Functional analyses further reveal that specific crotonylation-regulated targets modulate tumor proliferation, migration, and immune sensitivity; nonetheless, *in vivo* mechanistic validation is still absent (58). Overall, the interaction between tumor crotonylation and the immune microenvironment (or immune response) displays marked cancer-type- and context-specific characteristics.

### 2.4 Role of crotonylation in tumors

#### 2.4.1 Breast cancer

Breast cancer is one of the most prevalent malignant tumors in women, and its disease progression and chemosensitivity are modulated by a multitude of factors. Among them, protein post-translational modification (PTM) is an important regulatory mechanism (59). Lysine crotonylation, a recently recognized and functionally significant PTM, is increasingly recognized as a key driver in diverse biological processes underlying breast cancer pathogenesis, including metabolic reprogramming, tumor progression, immune regulation, and therapy resistance (**Figure 2**).

**Figure 2:**
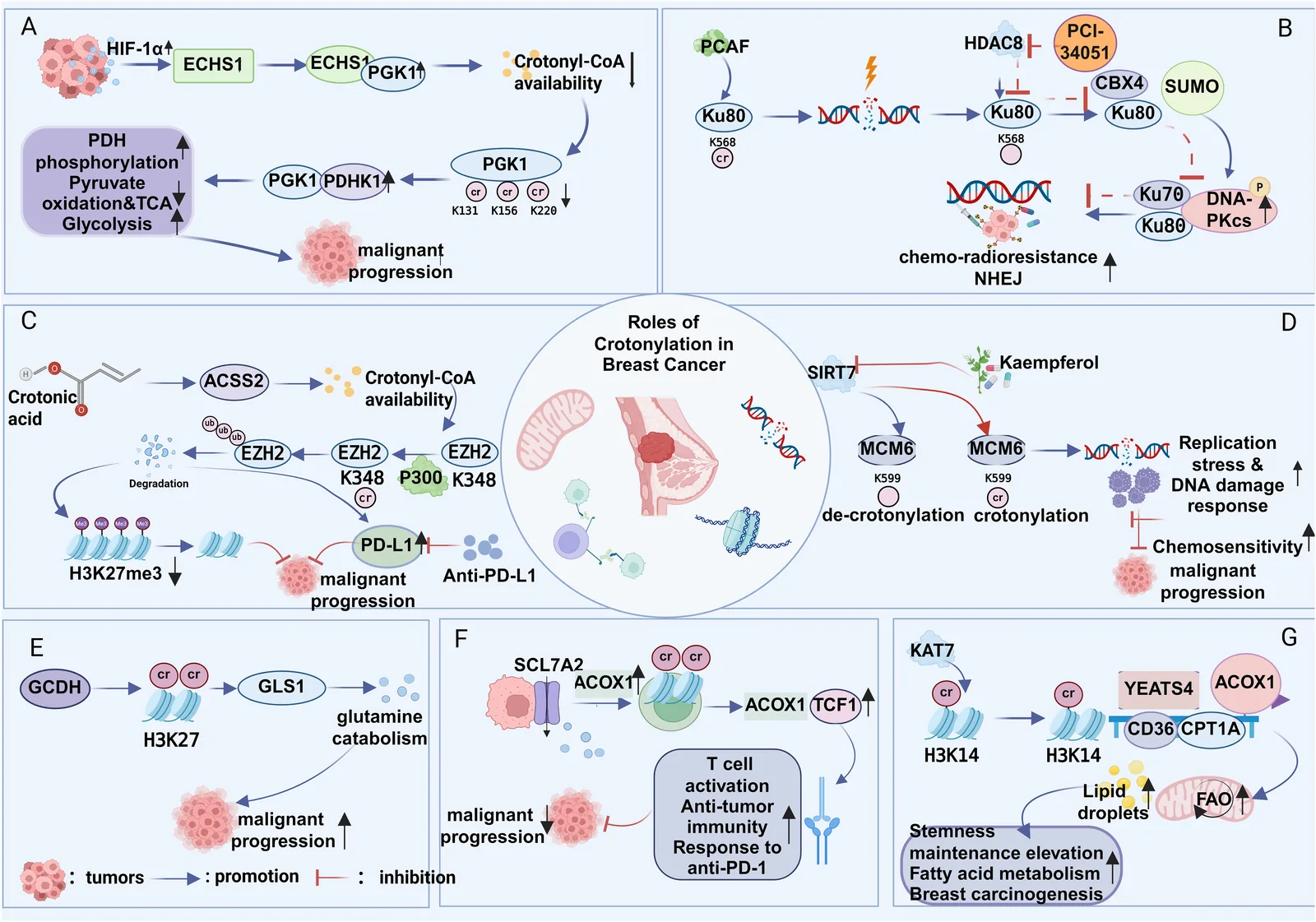
Proposed mechanisms of crotonylation in breast cancer.This schematic integrates findings from multiple studies. All panels refer to breast cancer; panel F additionally involves T cells. **(A)** Hypoxia-induced HIF-1α upregulates ECHS1, which reduces PGK1 crotonylation at K131/K156/K220, increasing PDH phosphorylation, decreasing pyruvate oxidation and TCA cycle, enhancing glycolysis, and promoting malignant progression. **(B)** DNA damage induces PCAF-mediated Ku80 K568 crotonylation, which is removed by HDAC8 (inhibited by PCI-34051), promoting CBX4-dependent SUMOylation, DNA-PKcs activation, NHEJ repair, and chemo-radioresistance. **(C)** Crotonic acid is converted by ACSS2 to crotonyl-CoA, promoting p300-mediated EZH2 K348 crotonylation, EZH2 degradation, H3K27me3 decrease, PD-L1 increase, and malignant progression; anti-PD-L1 may counteract this. **(D)** SIRT7 de-crotonylates MCM6 K599; kaempferol inhibits SIRT7, increasing MCM6 K599 crotonylation, replication stress, DNA damage response, and chemosensitivity. **(E)** GCDH promotes H3K27 crotonylation at the GLS1 promoter, increasing glutamine catabolism and malignant progression. **(F)** SLC7A2 upregulates ACOX1, increasing TCF1 in T cells, promoting T cell activation, anti-tumor immunity, and response to anti-PD-1, while suppressing malignant progression. **(G)** KAT7-mediated H3K14 crotonylation is recognized by YEATS4, activating lipid metabolism genes (CD36, CPT1A, ACOX1), promoting FAO, lipid droplet accumulation, stemness, and breast cancer progression. Blue arrows: promotion/activation; red T-bars: inhibition.

Dysregulation of lysine crotonylation is strongly associated with breast cancer initiation, progression and clinical prognosis, establishing it as a central epigenetic target in breast cancer research. Within the hypoxic microenvironment characteristic of breast cancer, hypoxia-inducible factor-1α (HIF-1α) transcriptionally upregulates the expression of enoyl-CoA hydratase short chain 1 (ECHS1), thereby strengthening its physical interaction with phosphoglycerate kinase 1 (PGK1). This enhanced association impairs the cytosolic and mitochondrial utilization of crotonyl-CoA, resulting in reduced crotonylation of PGK1 at lysine residues K131cr, K156cr, and K220cr. Subsequently, decrotonylation facilitates PGK1’s functional coupling with the pyruvate dehydrogenase kinase 1 (PDHK1) signaling axis, augmenting PDHK1-mediated inhibitory phosphorylation of pyruvate dehydrogenase (PDH). Consequently, mitochondrial pyruvate oxidation and tricarboxylic acid (TCA) cycle flux are suppressed, promoting metabolic reprogramming toward aerobic glycolysis. Clinical analyses of patient samples reveal that low levels of PGK1-K131cr crotonylation are significantly correlated with heightened tumor malignancy and poorer prognosis. Collectively, these findings establish site-specific crotonylation of PGK1 as a pivotal regulatory node that integrates hypoxic signaling, metabolic reprogramming, and malignant progression in breast cancer (6). Studies have confirmed that the p300-CBP-associated factor (PCAF), acting as an upstream modifying enzyme, specifically catalyzes crotonylation of Ku80 at lysine 568 (K568).Upon induction of DNA double-strand breaks (DSBs) in tumor cells, the decrotonylase HDAC8 mediates removal of the crotonyl moiety from Ku80 K568, thereby resetting this post-translational modification site and triggering CBX4-dependent SUMOylation of Ku80. This dynamic crotonylation–SUMOylation switch serves as a critical regulatory signal that facilitates assembly of the DNA-PK complex and promotes autophosphorylation of DNA-PKcs at serine 2056 (S2056), leading to full activation of the non-homologous end joining (NHEJ) repair pathway in tumor cells. Consequently, NHEJ repair capacity is enhanced, ultimately contributing to chemoradiotherapy resistance in malignant tumors. Treatment with the HDAC8 inhibitor PCI-34051 blocks Ku80 K568cr decrotonylation, thereby inhibiting DNA-PK complex assembly and DSB repair, ultimately achieving tumor radiosensitization (22). Liu et al. demonstrated that exogenous crotonate is converted to crotonyl-CoA by acyl-CoA synthetase short-chain family member 2 (ACSS2), which subsequently enables p300-mediated crotonylation of EZH2 at K348cr. This modification promotes EZH2 ubiquitination and proteasomal degradation, thereby alleviating H3K27me3-mediated transcriptional repression and suppressing breast cancer metastasis. Moreover, combination therapy with crotonate supplementation and anti-PD-L1 immunotherapy synergistically enhances antitumor effects, underscoring the multifaceted role of crotonylation in regulating tumor proliferation, metastasis, and immunotherapy sensitivity (9). YEATS4 is frequently overexpressed in breast cancer—particularly in estrogen receptor–positive (ER+) subtypes; the histone acetyltransferase KAT7 catalyzes histone crotonylation. As a key”reader”of histone H3 lysine 14 crotonylation (H3K14cr), YEATS4 binds to H3K14cr-marked chromatin and activates transcription of lipid metabolism–associated genes—including CD36, CPT1A, and ACOX1—thereby driving the PPARα-regulated fatty acid uptake–fatty acid oxidation (FAO) axis. This metabolic reprogramming results in increased fatty acid uptake, accumulation of intracellular lipid droplets, elevated oxygen consumption rate (OCR) and increased ATP production, and enhanced FAO flux. Ultimately, these changes promote the self-renewal of ALDH+ breast cancer stem cells and accelerate tumor growth. Clinically, high YEATS4 expression correlates significantly with poor patient prognosis (57). GCDH enhances glutamine catabolism by promoting H3K27cr crotonylation at the glutaminase 1 (GLS1) promoter, thereby upregulating GLS1 transcription and ultimately driving tumor cell proliferation—thus confirming a critical functional link between histone crotonylation and amino acid metabolism in tumorigenesis (60). In the context of tumor immunity, Sun et al. demonstrated that SLC7A2 upregulates ACOX1 expression in CD8+ T cell in triple-negative breast cancer, leading to increased histone crotonylation and enhanced physical interaction between ACOX1 and TCF1. This cascade potentiates anti-tumor T-cell immunity and improves the efficacy of immune checkpoint inhibitors (18). These findings corroborate prior evidence that exogenous crotonate augments immunotherapy sensitivity (9), underscoring the dual role of crotonylation: it suppresses intratumoral metastatic potential while simultaneously remodeling the immune microenvironment to strengthen therapeutic response. Crotonylation holds substantial translational relevance in modulating tumor drug resistance and represents a pivotal frontier in current mechanistic studies of this phenomenon. Song et al. identified the decrotonylase SIRT7 as a key regulator of MCM6 K599cr decrotonylation. Specifically, SIRT7 maintains low crotonylation levels at this residue to preserve genomic replication fidelity; conversely, elevated MCM6 K599cr crotonylation triggers replication stress and activates the DNA damage response, thereby sensitizing tumor cells to chemotherapeutic agents. Mechanistically, they further showed that kaempferol effectively inhibits SIRT7 activity, resulting in increased MCM6 crotonylation and synergistic anti-tumor effects when combined with paclitaxel. Collectively, these results support targeting decrotonylases to restore site-specific protein crotonylation as a promising strategy for chemotherapy sensitization (41). In summary, emerging research on crotonylation in breast cancer is advancing precision oncology, offering novel epigenetic targets and conceptual frameworks for next-generation epigenetic therapeutics.

#### 2.4.2 Colorectal cancer

Colorectal cancer is a highly prevalent malignant neoplasm worldwide, and its initiation and progression are governed by diverse molecular mechanisms—among which aberrant protein post-translational modifications (PTMs) play a critical role. Accumulating evidence indicates that lysine crotonylation, a recently characterized PTM, exerts pivotal regulatory functions in colorectal carcinogenesis and tumor progression (**Figure 3**). Notably, under hypoxic conditions, HIF-2α recruits the histone acetyltransferases p300/CBP to the autotaxin (ATX) promoter region, thereby enhancing H3K18cr. This epigenetic activation triggers the ATX–lysophosphatidic acid (LPA) signaling axis, ultimately promoting colorectal cancer cell migration (61). In another mechanism, the long non-coding RNA LINC00922 functions as a molecular decoy for SIRT3, a NAD^+^-dependent decrotonylase, thereby sequestering SIRT3 away from the ETS1 promoter. Consequently, local H3K27cr crotonylation levels increase, leading to transcriptional activation of ETS1 and subsequent upregulation of cell adhesion molecules (CAMs), which drive tumor invasion and metastasis (10). At the RNA level, a distinct regulatory paradigm has been identified: the lncRNA-like MMP14 transcript specifically interacts with the N-terminal domain of SIRT3, serving as a molecular scaffold that facilitates targeted recruitment of SIRT3 to the STARD13 promoter. There, SIRT3 catalyzes the removal of H3K27crotonylation, resulting in chromatin condensation and transcriptional repression of the tumor-suppressive gene STARD13, thereby facilitating colorectal cancer metastasis (13).

**Figure 3:**
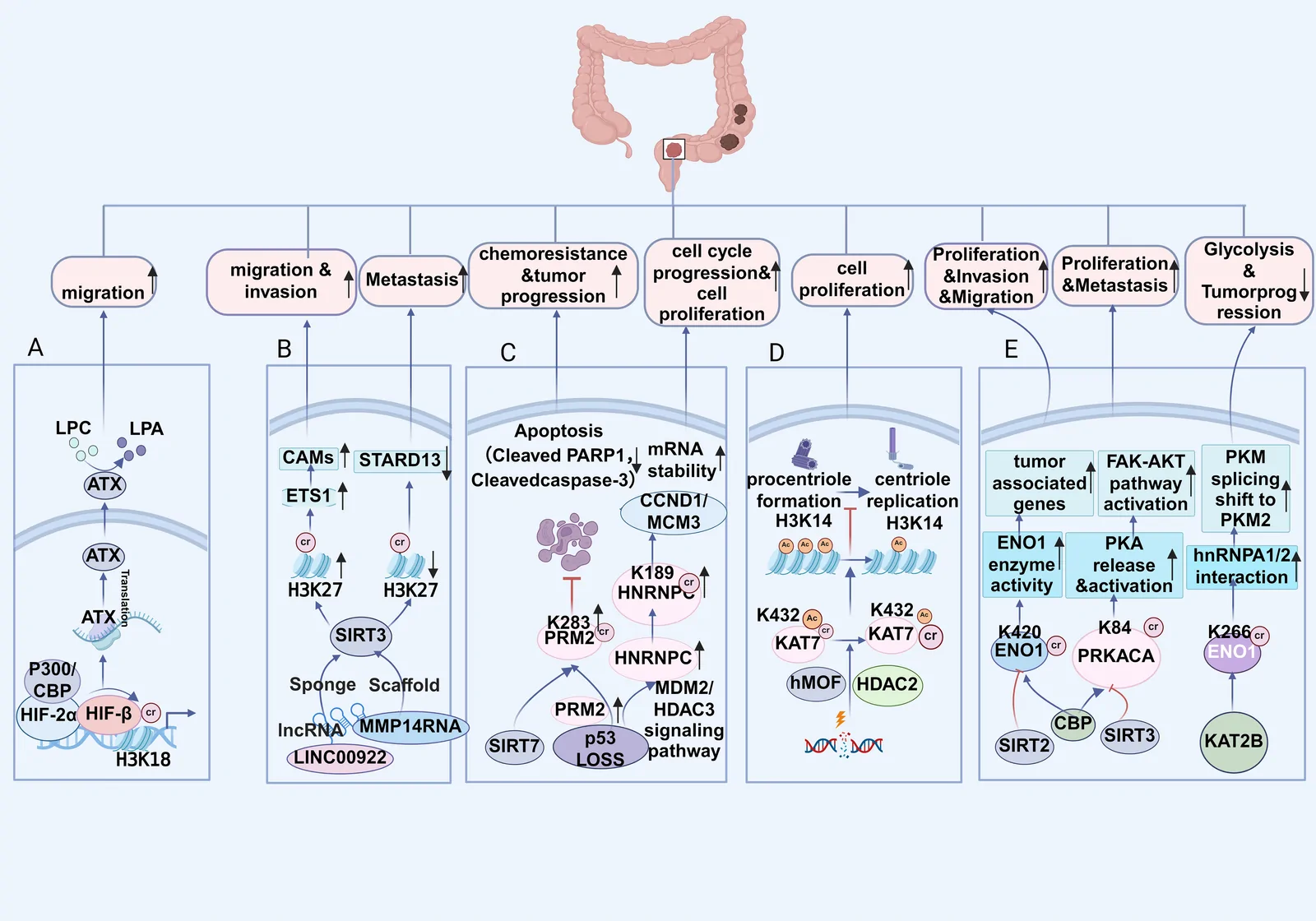
Proposed mechanisms of crotonylation in colorectal cancer.This schematic integrates findings from multiple studies. All panels refer to colorectal cancer cells. **(A)** Hypoxia-induced HIF-2α recruits p300/CBP to the ATX promoter, increasing H3K18cr and activating the ATX–LPA axis, promoting migration. **(B)** LINC00922 sequesters SIRT3, increasing H3K27cr at the ETS1 promoter and upregulating CAMs; MMP14 RNA scaffolds SIRT3 to the STARD13 promoter, reducing H3K27cr and repressing STARD13, promoting migration, invasion, and metastasis. **(C)** p53 loss upregulates RRM2 K283cr via MDM2/HDAC3 and HNRNPC K189cr, increasing CCND1/MCM3 mRNA stability, inhibiting apoptosis, and promoting chemoresistance and cell cycle progression. **(D)** hMOF-mediated KAT7 K432cr competes with HDAC2, altering H3K14ac and promoting procentriole formation and cell proliferation. **(E)** CBP-mediated ENO1 K420cr, PRKACA K84cr, and KAT2B-mediated PTBP1 K266cr enhance enzyme activity, PKA activation, and PKM2 splicing, promoting proliferation, invasion, migration, and metastasis; SIRT2/SIRT3 reverse these effects.Blue arrows: promotion/activation; red T-bars: inhibition; cr: crotonylation.

These studies collectively demonstrate that both hypoxia and long non-coding RNAs (lncRNAs) function as upstream regulatory signals that modulate tumor progression through the regulation of protein crotonylation. Secondly, crotonylation can directly modulate the activity of metabolic enzymes and signaling proteins to influence tumorigenesis and cancer progression. For instance, crotonylation of α-enolase (ENO1) at K420cr is markedly upregulated in colorectal cancer. This modification is catalyzed by the crotonyltransferase CBP and reversed by the decrotonylase SIRT2. ENO1 crotonylation enhances its enzymatic activity and regulates the expression of downstream tumor-associated genes, thereby promoting the proliferation, migration, and invasion of colorectal cancer cells (14). Similarly, crotonylation of the cAMP-dependent protein kinase catalytic subunit alpha (PRKACA) at K84 is coordinately regulated by CBP and SIRT3. This post-translational modification attenuates the interaction between the catalytic and regulatory subunits of protein kinase A (PKA), leading to sustained dissociation and activation of PKA. Consequently, the downstream FAK–AKT signaling pathway is activated, driving tumor proliferation and metastasis (33).

Furthermore, KAT2B-mediated crotonylation of polypyrimidine tract-binding protein 1 (PTBP1) at K266cr strengthens its binding to heterogeneous nuclear ribonucleoproteins A1 and A2 (hnRNP A1/A2), thereby shifting the alternative splicing of pyruvate kinase M (PKM) toward the PKM2 isoform. This switch significantly augments glycolytic flux and enhances the tumorigenic potential of cancer cells (16). Furthermore, loss of the tumor suppressor p53 is also associated with lysine crotonylation. Specifically, p53 deletion concurrently upregulates both the expression of ribonucleotide reductase regulatory subunit M2 (RRM2) and its crotonylation at K283cr, thereby suppressing the activation of cleaved PARP1 and cleaved caspase-3 and ultimately inhibiting apoptosis. Concurrently, downregulation of SIRT7 impairs the decrotonylation of RRM2, contributing to cisplatin resistance and exerting pro-tumorigenic effects in colon cancer (15). Subsequent studies confirmed that p53 loss or functional impairment enhances HNRNPC expression and promotes its crotonylation at K189 via the MDM2/HDAC3 signaling axis, thereby increasing the mRNA stability of CCND1 and MCM3, sustaining aberrant cell cycle progression, and driving tumor proliferation (62). Collectively, these findings indicate that lysine crotonylation contributes not only to tumor cell metabolism and migration but also to chemotherapy resistance and dysregulated cell cycle maintenance downstream of p53 dysfunction. In contrast to the aforementioned mechanisms, Wang et al. proposed a model of “competitive antagonism” between lysine crotonylation and acetylation. Specifically, upon DNA damage, KAT7 is crotonylated by hMOF at K432cr, which competes with HDAC2-mediated deacetylation, thereby modulating KAT7’s histone acetyltransferase activity, altering H3K14 acetylation levels, and regulating the expression of genes involved in procentriole formation, ultimately influencing colorectal cancer progression and clinical prognosis (11). This study elucidates the mechanistic basis by which a single protein can perform distinct biological functions under varying stress conditions. A clinical investigation revealed that H2BK12cr levels are significantly elevated in patients with cancer and strongly correlate with distant metastasis and disease stage. Notably, H2BK12cr demonstrates superior diagnostic performance compared with conventional carcinoembryonic antigen (CEA), indicating that peripheral blood H2BK12cr may serve as a minimally invasive, highly sensitive biomarker for both screening and prognostication in colorectal cancer (63). In summary, protein crotonylation in colorectal cancer is coordinately regulated by multiple signaling inputs—including the tumor microenvironment, DNA damage responses and non-coding RNAs. Through modulation of histone marks and functional protein states, crotonylation orchestrates key oncogenic processes such as tumor metabolism, proliferation, metastasis, and chemotherapy resistance. These findings underscore its potential as a clinically relevant diagnostic biomarker for colorectal cancer and establish a robust theoretical framework for deciphering the epigenetic mechanisms underlying this malignancy, thereby facilitating the design of novel theranostic strategies.

#### 2.4.3 Liver cancer

Hepatocellular carcinoma (HCC) is a highly prevalent malignant tumor worldwide, characterized by an exceptionally poor overall prognosis and recognized as one of the most clinically aggressive cancer types (64). Accumulating evidence indicates that crotonylation is co-regulated during HCC development by the tumor microenvironment, metabolic substrate availability, and decrotonylase activity. Preclinical and early clinical studies have demonstrated that global crotonylation levels in HCC are strongly correlated with tumor stage (**Figure 4**). Notably, both the inhibition of HDAC1/3 and treatment with the pan-HDAC inhibitor trichostatin A (TSA) which elevates cellular crotonylation, significantly suppress the migration and proliferation of hepatocellular carcinoma cells. These findings suggest that crotonylation may exert tumor-suppressive functions under specific microenvironmental conditions (65). Under hypoxic conditions, tumor cells downregulate HDAC6 expression, thereby attenuating its decrotonylase activity toward lamin A. This leads to enhanced crotonylation of lamin A at lysine residues K265cr and K270cr. This site-specific modification stabilizes the subcellular localization of lamin A, promoting hepatocellular carcinoma cell proliferation and inhibiting cellular senescence. Collectively, these results provide novel insights into the regulation mediated by non-histone crotonylation, deepening our understanding of how hypoxia promotes malignant phenotypes in HCC (23). SIRT2 functions as a decrotonylase that regulates the crotonylation of septin 2 (SEPT2). Specifically, crotonylation of SEPT2 at K74cr maintains its GTPase activity and promotes liver cancer cell invasion and metastasis by activating the downstream P85α–AKT signaling pathway. Clinical analyses further demonstrate that elevated SEPT2-K74cr crotonylation levels correlate significantly with poor patient prognosis and increased postoperative recurrence, thereby identifying it as an independent prognostic biomarker (20). GCDH catalyzes the specific conversion of glutaryl-CoA to crotonyl-CoA, thereby directly regulating the cellular availability of the essential crotonylation substrate. GCDH upregulation enhances crotonylation on key metabolic enzymes, including PGD-K163cr, TKT-K140cr, and ALDOC-K28cr, leading to the suppression of the activity of rate-limiting enzymes in both the pentose phosphate pathway and glycolysis.This metabolic reprogramming shifts cellular redox homeostasis toward reactive oxygen species (ROS) accumulation, ultimately triggering tumor cell senescence, the establishment of the senescence-associated secretory phenotype (SASP), and the remodeling of the tumor immune microenvironment (46). Integrated multi-omics analyses of diverse post-translational modifications reveal that lysine crotonylation acts in concert with phosphorylation and acetylation to cooperatively regulate core oncogenic pathways including p53/MYC, PI3K–AKT–mTOR and antigen presentation. The KAT8/HAT1 and HDAC/SIRT enzyme families constitute the primary “writer–eraser” machinery for crotonylation in hepatocellular carcinoma, thus establishing a mechanistic foundation for rational combination-targeted therapies (66). A recent study that developed a predictive model for hepatitis B virus–related hepatocellular carcinoma (HBV-HCC) identified two distinct molecular subgroups characterized by differentially expressed lysine crotonylation–associated genes—thereby identifying promising biomarkers for risk stratification and potential targets for immunotherapeutic intervention (67). In summary, the regulatory mechanisms and disease-associated molecular markers associated with lysine crotonylation in liver cancer collectively reveal novel avenues for precision diagnosis and targeted therapeutic interventions.

**Figure 4:**
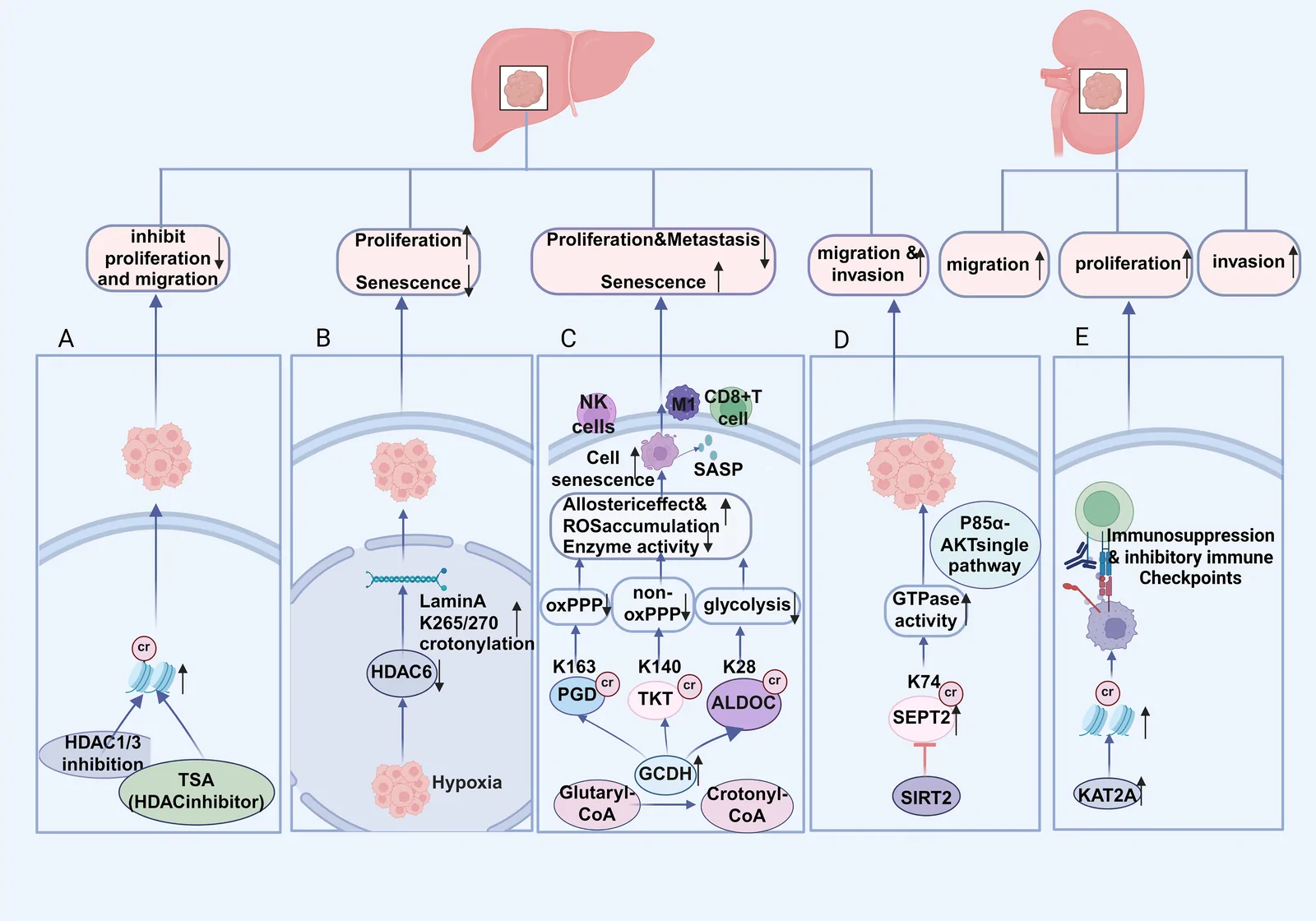
Proposed mechanisms of crotonylation in liver cancer and clear cell renal cell carcinoma. Panels **(A–D)** refer to liver cancer; **(E)** refers to clear cell renal cell carcinoma. **(A)** HDAC1/3 inhibition or TSA increases crotonylation, inhibiting proliferation and migration. **(B)** Hypoxia downregulates HDAC6, increasing lamin A K265/K270 crotonylation and promoting proliferation and senescence. **(C)** GCDH converts glutaryl-CoA to crotonyl-CoA, increasing PGD K163cr, TKT K140cr, and ALDOC K28cr, altering enzyme activity, ROS accumulation, and glycolysis, leading to SASP and proliferation/metastasis. **(D)** SIRT2 regulates SEPT2 K74cr, activating P85α-AKT signaling and promoting migration and invasion. **(E)** KAT2A-mediated crotonylation promotes proliferation, migration, invasion, and immunosuppression. Blue arrows: promotion/activation; red T-bars: inhibition; cr: crotonylation.

#### 2.4.4 Clear cell renal cell carcinoma

Renal cell carcinoma (RCC) is one of the most common malignancies of the urinary system worldwide. RCC includes several histopathological subtypes, such as clear cell RCC (ccRCC), papillary RCC (pRCC), and chromophobe RCC (chRCC) (68). Among these subtypes, ccRCC is the most common, accounting for approximately 70% of all RCC cases (69). Recent studies have shown that the crotonyltransferase KAT2A is significantly upregulated in ccRCC tissues. In ccRCC cell lines, KAT2A knockdown suppressed proliferation, migration, and invasion *in vitro*, providing genetic evidence for a pro-tumorigenic role of KAT2A (70). Moreover, elevated KAT2A expression was associated with an immunosuppressive tumor microenvironment in bioinformatics analyses, suggesting a potential link between KAT2A-mediated crotonylation and immune evasion (70). Based on crotonylation modification signatures, a prognostic risk model has been constructed for risk stratification in ccRCC patients. Further computational analyses suggest that intertumoral heterogeneity in crotonylation patterns is associated with key oncogenic processes, including immune evasion, tumor mutational burden, and metabolic reprogramming; however, these findings remain bioinformatics-derived and require experimental validation (71) (**Figure 4**).

#### 2.4.5 Pancreatic ductal adenocarcinoma

Pancreatic ductal adenocarcinoma (PDAC) ranks among the most lethal malignancies worldwide (64), with a poor 5-year survival rate of approximately 13%. Its incidence is significantly elevated in developed countries relative to developing regions. The molecular pathogenesis of PDAC is highly intricate, involving widespread dysregulation of multiple signaling cascades and metabolic reprogramming. The crotonylation, as a key regulator in metabolic reprogramming, holds critical importance for the development of therapeutic approaches for PDAC (**Figure 5**). Zheng et al. identified 4, 187 crotonylation sites across 1, 533 unique proteins, revealing significant enrichment in core metabolic pathways, including glycolysis, amino acid metabolism, fatty acid metabolism, and the tricarboxylic acid (TCA) cycle. Notably, methylenetetrahydrofolate dehydrogenase 1 (MTHFD1), the crotonylation of which is co-regulated by the acetyltransferases CBP/p300 and the deacetylases HDAC1/3, undergoes site-specific decrotonylation at lysine residues K354cr and K553cr. This site-specific decrotonylation activates MTHFD1, thereby suppressing ferroptosis and facilitating malignant progression (3). A recent seminal study demonstrated that GCDH and CBP cooperatively regulate crotonylation of acetyl-CoA acetyltransferase 2 (ACAT2) at K235cr. This modification promotes sterol regulatory element-binding protein 2 (SREBP2) activation, thereby promoting cholesterol biosynthesis while simultaneously attenuating endoplasmic reticulum stress and apoptosis—ultimately enhancing tumor proliferation and migration (17). Collectively, current evidence indicates that crotonylation preferentially targets critical lysine residues on key metabolic enzymes, thereby modulating central cellular processes such as redox homeostasis, ferroptosis, and lipid biosynthesis. These regulatory effects highlight its indispensable role in the malignant progression of pancreatic cancer.

**Figure 5:**
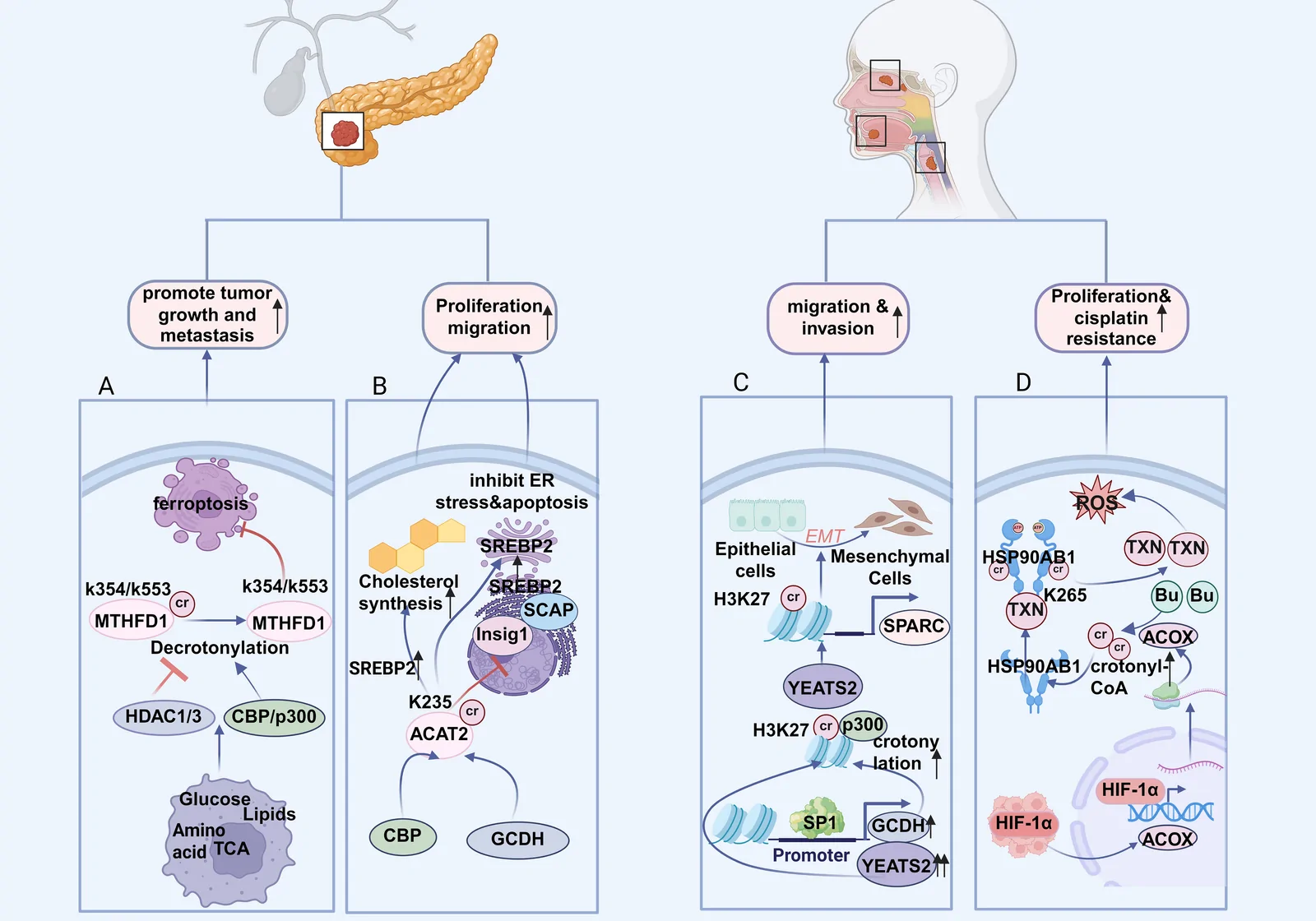
Proposed mechanisms of crotonylation in pancreatic ductal adenocarcinoma, Head and Neck Cancer &Esophageal Squamous Cell Carcinoma. Panels **(A, B)** refer to pancreatic ductal adenocarcinoma (PDAC); **(C, D)** refer to head and neck cancer (HNC)/esophageal squamous cell carcinoma (ESCC). **(A)** MTHFD1 K354/K553 crotonylation is regulated by HDAC1/3 (de-crotonylation) and CBP/p300 (crotonylation). MTHFD1 crotonylation suppresses ferroptosis and promotes tumor growth and metastasis, affecting glucose, lipid, and amino acid metabolism and the TCA cycle. **(B)** GCDH and CBP cooperatively regulate ACAT2 K235 crotonylation. ACAT2 K235cr activates SREBP2, promoting cholesterol synthesis, inhibiting ER stress and apoptosis, and enhancing proliferation and migration. **(C)** YEATS2 and p300 recognize H3K27cr to activate the SPARC promoter, while GCDH promotes crotonyl-CoA synthesis. Together, they drive EMT, migration, and invasion. **(D)** HIF-1α transcriptionally upregulates ACOX1, increasing crotonyl-CoA and driving HSP90AB1 K265 crotonylation. HSP90AB1 K265cr stabilizes TXN, alleviates ROS, promotes proliferation, and confers cisplatin resistance.Blue arrows: promotion/activation; red T-bars: inhibition; cr: crotonylation.

#### 2.4.6 Head and neck cancer & esophageal squamous cell carcinoma

Head and neck cancer (HNC) is an umbrella term for malignant tumors arising in various anatomical subsites within the head and neck region, such as the oral cavity, pharynx, larynx, nasal cavity, thyroid gland and salivary glands. A bioinformatics study identified a nine-gene prognostic signature derived from crotonylation regulatory factors as a candidate tool for prognostic evaluation in patients with head and neck squamous cell carcinoma (HNSCC); however, this signature remains computationally derived and requires prospective clinical validation (72). In preclinical cell models, biochemical and genetic evidence demonstrated that the histone reader YEATS2 exerts oncogenic activity in an SP1-dependent manner: YEATS2 recruits the acetyltransferase p300 to sustain elevated H3K27cr at the SPARC promoter, and GCDH promotes crotonyl-CoA synthesis. Together, YEATS2 and GCDH activated an EMT-associated transcriptional program and promoted tumor cell invasion and migration *in vitro (*73). Furthermore, integrative multi-acylation profiling, a bioinformatics analysis, revealed that a molecular subgroup with high global acylation modification scores was associated with poor clinical prognosis and an immunosuppressive tumor microenvironment, suggesting a potential relationship between dysregulated crotonylation and immune remodeling (74). A two-sample Mendelian randomization study suggested a potential causal association between genetically predicted elevated expression of GCDH and ACSS2 and increased HNC susceptibility, whereas elevated DPF2 and HDAC7 expression was associated with reduced risk; these findings are genetic association evidence and require functional validation (75). In OSCC cells, hypoxia induced K265 crotonylation of HSP90AB1, and mutation of K265 impaired its glycolysis-promoting function, providing biochemical and genetic evidence for a site-specific role of HSP90AB1 K265cr in glycolytic regulation (76). Subsequent mechanistic and *in vivo* studies demonstrated that HIF-1α transcriptionally upregulates ACOX1, increasing crotonyl-CoA availability and driving site-specific crotonylation of HSP90AB1 at K265; this modification stabilizes TXN, alleviates oxidative stress, promotes tumor cell proliferation, and confers cisplatin resistance in preclinical models (77). Notably, pharmacological inhibition of ACOX1 or TXN synergized with cisplatin to suppress tumor growth *in vivo*, supporting the therapeutic potential of targeting this axis (77). Finally, a bioinformatics study of esophageal squamous cell carcinoma (ESCC) integrated multiple cohorts and identified four crotonylation-related genes (OSM, FABP3, MICB, and FAM189A2) as significantly associated with overall survival; a candidate prognostic risk model and nomogram were constructed, but these findings are computational and require experimental and clinical validation (78)(**Figure 5**).

#### 2.4.7 Glioma

Glioma is a primary tumor arising from glial cells in the brain and spinal cord. It is the most common primary malignant brain tumor, characterized by aggressive proliferation and pronounced invasiveness, which pose substantial therapeutic challenges (79). Histone lysine crotonylation has been implicated in linking tumor metabolic reprogramming and epigenetic immune suppression (**Figure 6**). In glioblastoma stem cells (GSCs), biochemical and genetic evidence demonstrated that lysine uptake mediated by the lysine transporter SLC7A2 promotes intracellular accumulation of crotonyl-CoA through upregulation of GCDH and concomitant downregulation of ECHS1. Within the nucleus, the GCDH–CBP complex catalyzes histone H4 crotonylation, which competitively inhibits H3K27 acetylation, silences retrotransposons, and attenuates the MDA5/cGAS-dependent type I interferon response. This cascade suppresses CD8+ T-cell infiltration and sustains GSC stemness. Notably, in preclinical models, dietary lysine restriction combined with MYC inhibition reduced global histone crotonylation, reversed immunosuppression, and synergized with anti-PD-1 immunotherapy to inhibit tumor growth (19). These findings provide a mechanistic rationale and identify a candidate target for integrated metabolic–immune interventions in malignant gliomas. In a bioinformatics study, Yin et al. developed a crotonylation-associated prognostic risk model for glioma, in which high-risk patients were characterized by increased somatic mutations, specific copy number variations, and an immunosuppressive tumor microenvironment, with risk scores positively correlating with TIDE scores. Single-cell analysis suggested a positive association between the risk score and tumor-associated macrophage (TAM) infiltration. ChIP-seq and qPCR in T98G cells indicated that crotonate treatment reduced CXCL1 expression, and tissue staining showed that PD-1 was negatively, whereas GZMB was positively, correlated with crotonylation levels (80). These findings are primarily correlative and computational, and they identify candidate mechanisms linking crotonylation to the glioma immune microenvironment, pending further functional validation. In a separate study, Song et al. identified six lysine crotonylation-related lncRNAs (LCRlncRNAs) and constructed a risk model that stratified glioma patients into high- and low-risk groups with distinct survival outcomes. The high-risk group exhibited increased regulatory T cells, M2 macrophages, and elevated immune checkpoint expression (PD-L1, CTLA4), and TIDE analysis indicated poor immunotherapy response (81). Functional experiments demonstrated that POLR2J4 knockdown suppressed glioma cell proliferation, migration, and invasion *in vitro*, and inhibited tumor growth in xenograft models. Mechanistically, POLR2J4 knockdown reduced the expression of drug resistance genes (ABCB1, ABCC1, BCL2), decreased serum IL-6 and TGF-β1 levels, and downregulated TGF-β1 and PD-L1 in tumor tissues (81). These findings provide experimental evidence linking a specific LCRlncRNA to glioma progression, chemoresistance, and immunosuppressive microenvironment remodeling. Collectively, targeting crotonylation—and its upstream regulators and downstream effectors—represents a candidate strategy to overcome current limitations in glioma immunotherapy, though further mechanistic and clinical validation is required.

**Figure 6:**
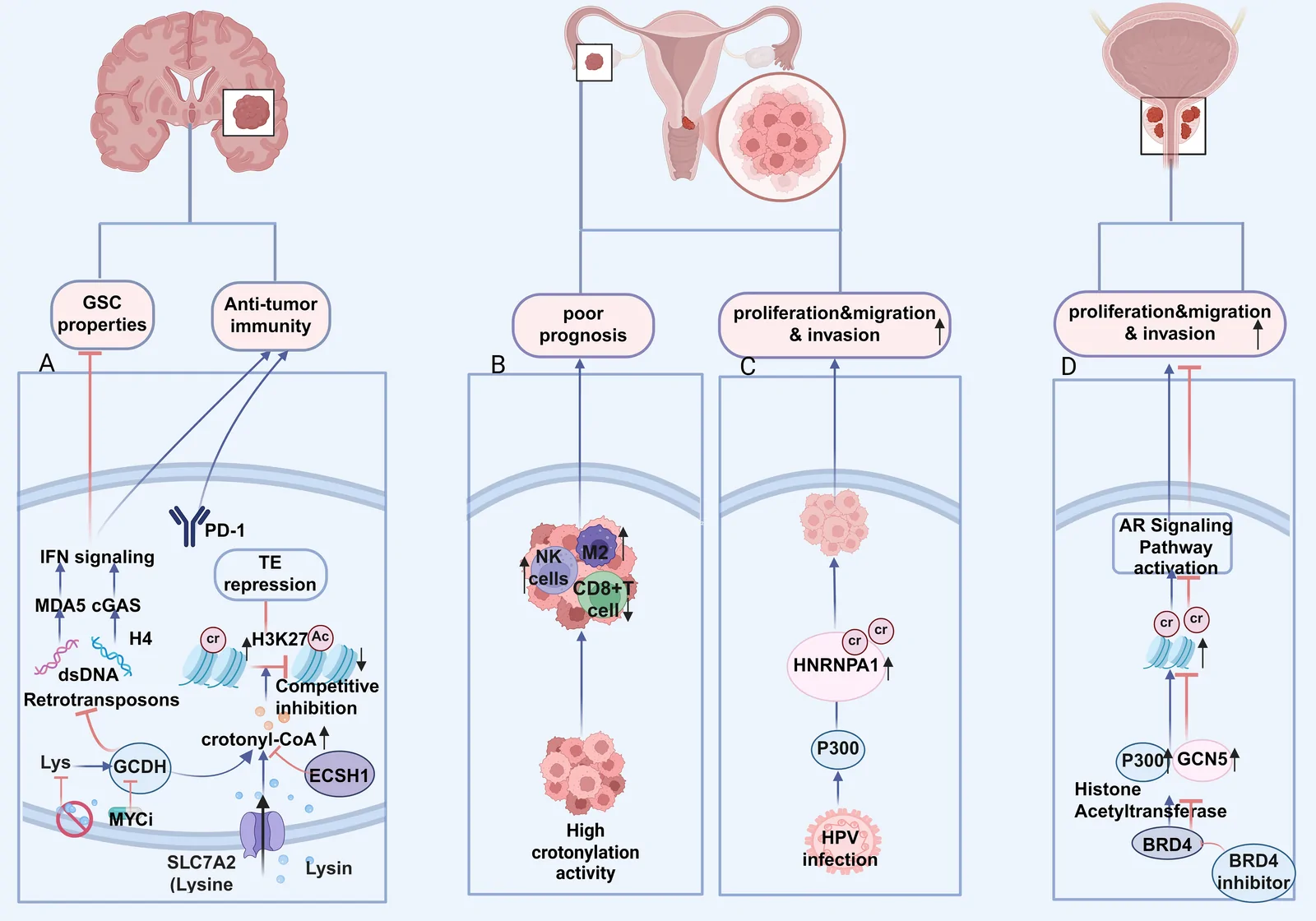
Proposed mechanisms of crotonylation in glioma, ovarian cancer, cervical cancer, and prostate cancer.This schematic integrates findings from multiple studies. **(A–D)** refer to glioma **(A)**, ovarian cancer **(B)**, cervical cancer **(C)**, and prostate cancer **(D)**. **(A)** In glioma stem cells, lysine (Lys) uptake via SLC7A2 promotes crotonyl-CoA accumulation through GCDH upregulation and ECHS1 downregulation. Increased histone H4 crotonylation (H4cr) competitively inhibits H3K27ac, represses TE (retrotransposons), attenuates MDA5/cGAS-type I interferon signaling, reduces CD8^+^ T cell infiltration, and sustains GSC properties. MYC inhibition and lysine restriction reverse these effects. **(B)** In ovarian cancer, high crotonylation activity is associated with decreased NK cells and CD8^+^ T cells, increased M2 macrophages, and poor prognosis. **(C)** In cervical cancer, HPV infection upregulates P300, which mediates HNRNPA1 crotonylation, promoting proliferation, migration, and invasion. **(D)** In prostate cancer, BRD4 regulates P300/GCN5-mediated histone crotonylation, activating AR signaling and promoting proliferation, migration, and invasion; BRD4 inhibitors block this axis. Blue arrows indicate promotion/activation; red T-bars indicate inhibition; cr denotes crotonylation.

#### 2.4.8 Cervical cancer

Cervical carcinogenesis is primarily attributed to persistent infections with high-risk human papillomavirus (HPV) strains (82), making it a leading cause of cancer-related mortality among women worldwide (83). To date, research on lysine crotonylation in cervical cancer remains extremely limited. Emerging evidence indicates that heterogeneous nuclear ribonucleoprotein A1 (HNRNPA1) is markedly overexpressed in cervical cancer cell lines (including HeLa, CaSki, and SiHa), with the most pronounced upregulation observed in HeLa cells. Notably, p300-catalyzed crotonylation of HNRNPA1 enhances its protein stability and expression levels, thereby facilitating tumor cell proliferation, invasion, and metastasis. These findings reveal a novel crotonylation-dependent oncogenic mechanism and identify crotonylation modulation as a promising therapeutic target for cervical cancer intervention (84). Although sirtuins SIRT2 (85) and SIRT3 (32) have been implicated in the regulation of cervical cancer progression, their roles have not been linked to crotonylation-specific functions. Thus, investigations into the functional significance, regulatory enzymes, and therapeutic potential of crotonylation in cervical cancer warrant rigorous future investigation (**Figure 6**).

#### 2.4.9 Ovarian cancer

Ovarian cancer is the most lethal gynecologic malignancy worldwide (86). For ovarian cancer, computational evidence derived from crotonylation-associated transcriptional signatures suggests that aberrant histone crotonylation may accompany remodeling of the tumor immune microenvironment and poorer clinical outcome (87). Li et al. jointly interrogated single-cell and bulk transcriptomic profiles and resolved considerable variation in crotonylation activity among cell subpopulations. Their training (discovery) set combined TCGA-OV tumor samples with GTEx normal ovarian controls after batch correction; external testing used four independent GEO cohorts (GSE140082, GSE26193, GSE26712, and GSE63885) (87). A prognostic risk signature was built from crotonylation-related genes prioritized through differential expression analysis, WGCNA, and univariate Cox regression, then fitted using an ensemble machine-learning strategy with 10-fold cross-validation (87). CIBERSORT-based deconvolution was applied to estimate immune infiltration, ESTIMATE to quantify stromal and immune scores, and TIDE to assess immune escape potential (87). The reported declines in follicular helper T cells and activated NK cells, and the increase in M2 macrophages, are therefore computational inferences from bulk expression data, not direct measurements of lysine crotonylation (Kcr) activity, and cannot serve as experimental evidence of a causal immune mechanism. Although validation across GEO cohorts demonstrates that the signature is robust and reproducible, biological causality remains unproven. Functional work is needed to determine whether dysregulated histone crotonylation contributes to immunosuppression (87) (**Figure 6**).

#### 2.4.10 Prostate cancer

Prostate cancer is a highly prevalent malignant neoplasm that substantially affects the health of the male genitourinary system, particularly in older men. The incidence of this disease has increased markedly in recent years, representing a serious threat to male morbidity and mortality (88). Histone lysine crotonylation is aberrantly upregulated in prostate cancer tissues, and its modification level correlates positively with tumor grade and malignancy. Bromodomain-containing protein 4 (BRD4) indirectly modulates histone crotonylation by regulating the expression of the histone acetyltransferases GCN5 and p300; This enhances activation of the androgen receptor signaling pathway, thereby promoting proliferation, migration and invasion in prostate cancer cell. BRD4 inhibitors effectively suppress histone crotonylation and impede androgen receptor pathway activation, exerting robust antitumor effects in both hormone-sensitive and castration-resistant prostate cancer models. These findings suggest that the histone crotonylation–BRD4 regulatory axis is a promising therapeutic target for prostate cancer (89)(**Figure 6**).

#### 2.4.11 Lung cancer

Lung cancer is primarily classified into non-small cell lung cancer (NSCLC) and small cell lung cancer (SCLC). NSCLC comprises major histological subtypes, namely lung adenocarcinoma (LUAD) and lung squamous cell carcinoma (LUSC). Xu et al. identified, through global profiling of non-histone proteins in H1299 cells, 2, 696 lysine crotonylation sites across 1, 024 proteins, revealing that non-histone crotonylation is widespread and involves enzymes such as CBP, PCAF, MOF, HDAC1, and HDAC3 that can catalyze crotonylation or decrotonylation (39). Multiple experimental studies have shown that BEX2 is specifically crotonylated at lysine 59 under chemotherapeutic treatment or cellular stress. This modification facilitates BEX2 binding to NDP52, promotes their co-localization on damaged mitochondrial membranes, and strengthens the interaction between BEX2 and LC3B, thereby activating mitophagy and attenuating chemotherapy-induced apoptosis in preclinical models. This crotonylation-mediated pathway enhances lung cancer cell survival and is clinically associated with poor prognosis in LUAD (21). In EGFR-TKI-resistant cells, dysregulation of intracellular pH downregulates ACSS2 expression, reducing crotonyl-CoA production and causing a marked decrease in histone H3K56cr. Reduced H3K56cr suppresses HNF1A transcription, leading to PTEN downregulation and sustained PI3K/AKT hyperactivation. Notably, in preclinical models including cell lines, CDX, PDX, and PDO, the histone decrotonylase inhibitor S67 restored H3K56cr levels, reactivated HNF1A, inhibited AKT signaling, and reversed resistance (12). By combining WGCNA, machine learning, cellular functional experiments, and clinical sample verification, multiple studies have identified GAPDH, SLC25A13, MMACHC, and HDAC1 as crotonylation-related signature genes that are overexpressed in LUAD and associated with poor prognosis. These genes were computationally associated with immune cell infiltration and immune-inflammatory pathways. Among them, MMACHC knockdown inhibited proliferation and migration, induced apoptosis, and enhanced immunotherapy efficacy in LUAD cells (58). A recent investigation reported that FAM83A regulates H3K27cr and upregulates transcription of PKM2 and LDHA, promoting glycolysis and proliferation in lung cancer cells. Furthermore, a prognostic risk model derived from crotonylation-associated genes was associated with clinical prognosis; high-risk individuals exhibited higher tumor burden and a more immunosuppressive tumor microenvironment (90). These findings provide candidate biomarkers and potential therapeutic targets for LUAD (**Figure 7**).

**Figure 7:**
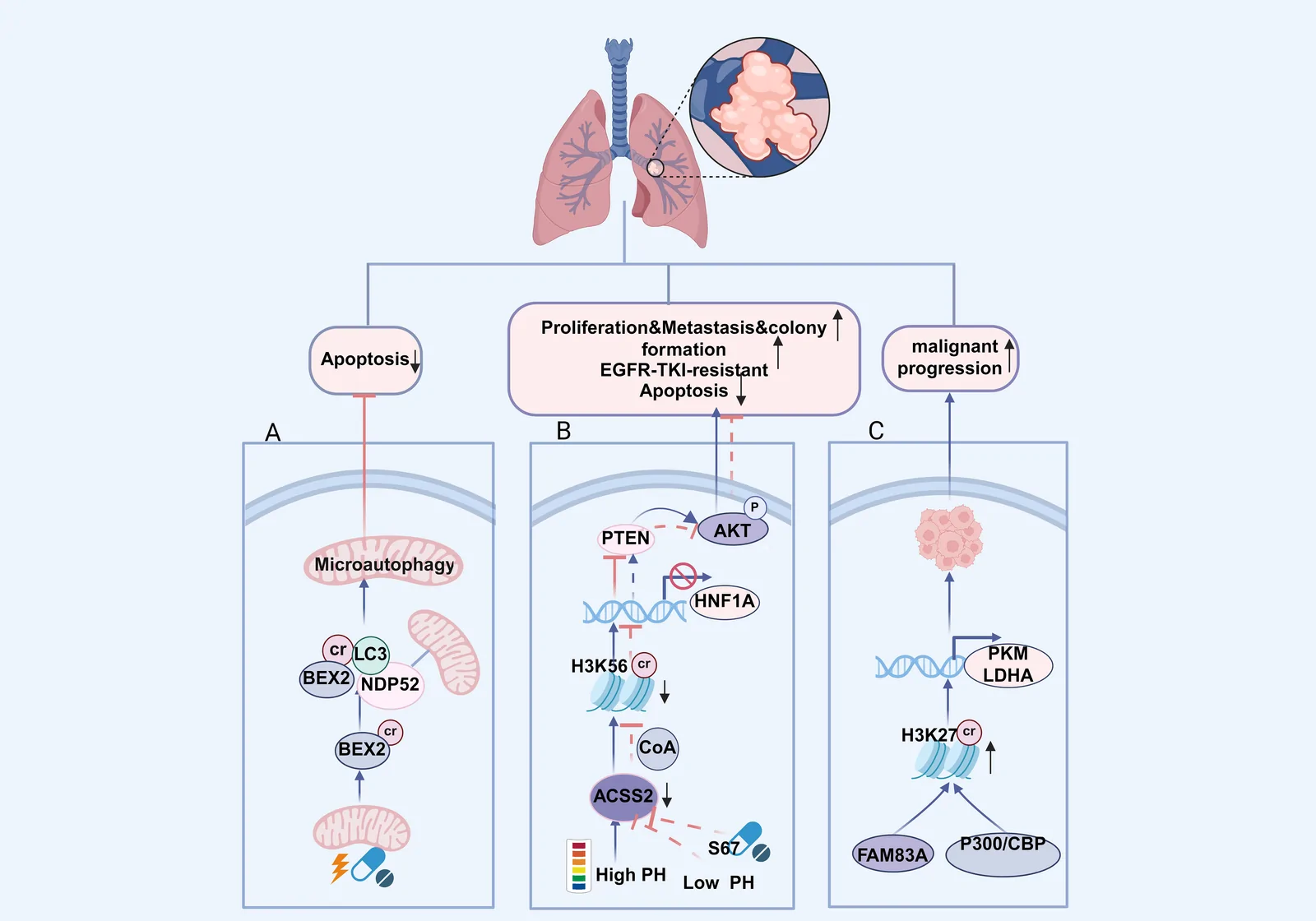
Proposed mechanisms of crotonylation in lung cancer.This schematic integrates findings from multiple studies. All panels refer to lung cancer cells, primarily lung adenocarcinoma (LUAD) and EGFR-TKI–resistant models. **(A)** Crotonylated BEX2 (BEX2 cr) interacts with NDP52 and LC3, activating mitophagy and attenuating chemotherapy-induced apoptosis. **(B)** In EGFR-TKI–resistant cells, low intracellular pH downregulates ACSS2, reducing crotonyl-CoA and H3K56cr. This suppresses HNF1A transcription, leading to PTEN downregulation and sustained AKT activation. The decrotonylase inhibitor S67 restores H3K56cr, reactivates HNF1A, inhibits AKT, and reverses resistance. **(C)** FAM83A and P300/CBP increase H3K27cr, upregulating PKM and LDHA transcription, promoting glycolysis and malignant progression.Blue arrows indicate promotion/activation; red T-bars indicate inhibition; cr denotes crotonylation.

#### 2.4.12 Thyroid cancer

Thyroid cancer is the most common endocrine malignancy, with papillary thyroid carcinoma (PTC) serving as its predominant histopathological subtype, which accounts for over 90% of all cases (91, 92). A recent investigation, combining mass spectrometry and bioinformatics, pinpointed 262 differentially crotonylated proteins and 702 crotonylation sites within thyroid cancer tissues. The results demonstrated a marked elevation in protein crotonylation levels during tumor advancement and lymph node metastasis, with these modifications functionally governing oncogenic mechanisms via critical signaling and metabolic pathways, such as PI3K–Akt, Hippo, cell cycle control, pyruvate metabolism and the tricarboxylic acid (TCA) cycle. Notably, crotonylation is markedly upregulated in tumor tissues and exhibits a strong positive correlation with N-cadherin expression; moreover, crotonylation-associated proteins serve as potential biomarkers predictive of poor patient prognosis (8). This study establishes a crucial foundation for subsequent mechanistic inquiries into the role of crotonylation in thyroid carcinogenesis. To date, investigations into crotonylation within the context of thyroid cancer have been limited, with the majority being restricted to bioinformatics-driven explorations.Thus, subsequent studies should prioritize elucidating underlying molecular mechanisms and advancing translational applications toward clinical utility.

Collectively, the studies summarized in this section do not support a single, unidirectional role for lysine crotonylation in cancer. Whether Kcr restrains or accelerates malignancy is determined by the modified substrate and site, the configuration of writers, erasers and readers, the availability and metabolic origin of crotonyl-CoA, and the cellular and immune context. Several principles are nevertheless reproducible across models: Kcr is site-specific and reversible; p300/CBP, KAT2A, KAT7, KAT8/hMOF and PCAF recur as writers, whereas HDAC1/3/6/8 and SIRT2/3/7 recur as erasers; GCDH-dependent crotonyl-CoA supply links lysine and glutaryl-CoA metabolism to chromatin and non-histone Kcr; and Kcr regulates core metabolic enzymes in glycolysis, glutaminolysis, fatty acid oxidation, cholesterol biosynthesis and the TCA cycle. By contrast, directionality remains controversial—global Kcr elevation can suppress hepatocellular carcinoma migration yet SIRT2-mediated SEPT2 decrotonylation promotes liver cancer invasion, and Kcr can enhance CD8^+^ T-cell immunity in triple-negative breast cancer while sustaining immune evasion in glioblastoma stem cells. Many reported mechanisms, biomarkers and risk models still rest on single studies or on computational inference from bulk or single-cell transcriptomes rather than direct Kcr measurement, and selective Kcr-directed tools and prospective human validation remain largely lacking. Resolving these open questions will require site-specific antibodies, quantitative Kcr proteomics, chemical probes, conditional knockout models, patient-derived organoids and prospective clinical cohorts, as discussed in the Conclusion.

### 2.5 Therapeutic strategies and application prospects of crotonylation

#### 2.5.1 Therapeutic strategies

Protein crotonylation has emerged as a critical epigenetic regulator in tumorigenesis and progression. However, no crotonylation-targeting therapeutic has been approved for cancer, and all agents discussed in this section remain preclinical. These agents fall into two mechanistically distinct categories: those that directly target the crotonylation machinery, and those that alter crotonylation indirectly as one downstream consequence among many.

Direct Kcr machinery-targeting agents include S67, a reported HDAC1-targeting decrotonylase inhibitor that elevates Kcr by blocking decrotonylation; it remains preclinical, and no clinical trials have been reported (12). Kaempferol inhibits SIRT7, thereby increasing MCM6 K599 crotonylation and inducing replication stress and DNA damage; the kaempferol and paclitaxel combination is likewise preclinical (41). However, kaempferol is not Kcr-selective and has multiple targets. PCI-34051, a selective HDAC8 inhibitor, acts on the Kcr eraser machinery by preventing decrotonylation at Ku80 K568cr. In preclinical models, this blockade impaired DNA-PK complex assembly and DSB repair, thereby enhancing radiosensitivity.Nevertheless, this compound remains at the preclinical stage, and no clinical evaluation has yet been reported (22). In the substrate-precursor category, crotonate is not the direct acyl donor for Kcr; rather, crotonyl-CoA serves as the acyl donor, and crotonate must first be converted to crotonyl-CoA, primarily by ACSS2. Crotonate supplementation increases EZH2-K348cr crotonylation, promotes EZH2 degradation, suppresses breast cancer invasion and metastasis, and synergizes with anti-PD-L1 in preclinical models (9). However, pharmacokinetics, oral bioavailability, and safety were not reported in that study. Crotonate should therefore be regarded as a preclinical substrate-precursor strategy rather than a clinically validated agent.

By contrast, indirect Kcr-modulating agents alter Kcr as a downstream consequence of other primary targets. TSA, a broad-spectrum class I/II HDAC inhibitor, has been reported to elevate histone crotonylation in HCC models. However, the research mainly verified the effects of HDAC inhibition or TSA on HCC cell migration and proliferation and does not provide direct evidence that TSA-induced histone Kcr mediates a therapeutic strategy in liver cancer (65). TSA is therefore considered an indirect Kcr-modulating agent with pleiotropic effects rather than a Kcr-directed therapeutic. Similarly, BRD4 inhibitors, including I-BET762, I-BET726, and CPI-203, do not directly target Kcr; they suppress BRD4, alter acetyltransferase expression, and indirectly reduce histone crotonylation. Their antitumor effects in prostate cancer models therefore do not constitute evidence for Kcr-directed therapy (89). Combination strategies including crotonate plus anti-PD-L1, kaempferol plus paclitaxel, and S67 plus EGFR-TKIs have shown preclinical promise but have not been clinically validated (**Table 2**).

**Table 2:** Therapeutic strategies and application prospects of crotonylation.

Agent	Mechanism of Kcr modulation	Selectivity	Tumor	Evidence stage	Principal safety limitation	Ref.
S67	Inhibits decrotonylation; increases Kcr	Reported HDAC1-selective; selectivity across other HDACs/acyl modifications not fully established	Lung cancer	Preclinical	Not established; potential off-target effects	(12)
Kaempferol	Inhibits SIRT7; increases MCM6 K599 crotonylation; replication stress/DNA damage; chemosensitization	Not Kcr-selective; multiple targets	Pan-cancer	Preclinical	Selectivity and safety not established; potential off-target effects	(41)
PCI-34051	Inhibits decrotonylation of Ku80 K568; impairs DNA-PK complex assembly and DSB repair; radiosensitization and chemosensitization	HDAC8-selective	Breast cancer	Preclinical	Not established; potential off-target effects	(22)
Crotonate	Increases crotonyl-CoA supply; EZH2 K348 crotonylation; EZH2 degradation; suppresses invasion/metastasis; enhances anti-PD-L1 in preclinical models	Substrate-level; no site selectivity	Breast cancer	Preclinical	Pharmacokinetics, oral bioavailability and safety not reported	(9)
TSA	Broad HDAC inhibition; reported to elevate histone Kcr in HCC models, but Kcr-mediated therapeutic effect not directly demonstrated	Broad; not Kcr-selective	Hepatocellular carcinoma	Preclinical	Pan-HDAC inhibition; pleiotropic effects; Kcr-independent mechanisms likely	(65)
BRD4 inhibitors (I-BET762, I-BET726, CPI-203)	Suppress BRD4; alter acetyltransferase expression; indirectly downregulate histone Kcr	BET-selective; not Kcr-directed	Prostate cancer	Preclinical	Pleiotropic BET inhibition; not Kcr-specific	(89)

#### 2.5.2 Application prospects

The global protein crotonylation level—or the crotonylation status at specific residues—shows potential as a biomarker for adjunctive clinical diagnosis, therapeutic response monitoring, and prognostic assessment in oncology. For instance, elevated crotonylation of KAT7 correlates with favorable prognosis in patients with colorectal cancer (11). Conversely, reduced crotonylation of PGK1-K131cr is associated with poor clinical outcomes in breast cancer patients (6). Moreover, histone H2B lysine 12 crotonylation levels in peripheral blood mononuclear cells (PBMCs) of colorectal cancer patients may serve as a biomarker to distinguish affected individuals from healthy controls (63). Collectively, these findings lay a critical foundation for the development of novel, crotonylation-based biomarkers in tumor diagnostics and management.

## 3 Conclusion

Lysine crotonylation, a pivotal post-translational modification that bridges cellular metabolism and epigenetic regulation, has emerged as a central focus in tumor biology research. Since its initial identification as a novel histone mark in 2011, the functional scope of Kcr has expanded beyond histones to encompass diverse non-histone substrates, including metabolic enzymes and DNA repair factors, which underscores its broad, multi-pathway regulatory roles. Kcr dynamics are governed by a canonical “writer–eraser–reader” enzymatic framework: “Writers” such as p300/CBP and MOF catalyze crotonyl group transfer; “erasers” such as class I histone deacetylases (HDACs) and SIRT1/2/3 mediate decrotonylation; and “readers” from the YEATS and DPF protein families recognize the modification and transduce it into context-specific biological outcomes. Critically, intracellular crotonyl-CoA, the direct acyl donor for Kcr, is tightly regulated by metabolic pathways such as fatty acid oxidation and lysine catabolism, positioning it as a key molecular nexus between cellular metabolism and epigenetic control. Crotonylation exerts critical biological functions through three core regulatory axes: in the transcriptional regulation, histone crotonylation is enriched in transcriptionally active chromatin regions and is strongly associated with the activation of oncogenes and other tumor-promoting genes, thereby facilitating malignant transformation. In the metabolic reprogramming, crotonylation post-translationally modifies key metabolic enzymes, particularly those involved in glycolysis and the TCA cycle, directly modulating their catalytic activity and consequently reshaping cellular metabolic flux. In the DNA damage response and immune regulation, crotonylation regulates pivotal DNA repair factors such as RPA1, thereby influencing the chemoradiosensitivity of tumor cells. Concurrently, it reshapes innate immune signaling pathways, including the cGAS-STING axis and modulates the infiltration levels of CD8^+^T cells, thereby participating in the shaping of the tumor immune microenvironment.

Although significant progress has been made in the research on crotonylation, there are still many challenges: First, the synergistic regulatory mechanisms governing crotonylation at specific sites, mediated by writers, erasers, and readers, remain poorly understood, and site-specific molecular tools for precise manipulation remain lacking. Second, the majority of current studies are restricted to preclinical models (e.g., cultured cells and animal systems), with limited validation in large-scale, well-annotated clinical cohorts; consequently, the translational relevance and clinical utility of crotonylation-associated targets remain uncertain. Third, extensive crosstalk between crotonylation and other post-translational modifications, including acetylation and methylation, hinders the design of selective therapeutic interventions targeting lysine crotonylation.

Furthermore, the development of small-molecule therapeutics targeting lysine crotonylation and the identification of biomarkers for patient stratification remain in the early stages. The field of high-resolution crotonyl proteomics is poised to accelerate the translation of lysine crotonylation research from fundamental mechanistic insights into clinical applications. Future initiatives should focus on three key areas: first, rigorously clarifying the site-specific functional roles of Kcr and their related regulatory networks to reduce off-target effects resulting from non-selective treatments; Second, rigorously validating the clinical utility of Kcr-related therapeutic targets and biomarkers in large-scale, multicenter clinical cohorts to establish cancer-type-specific prognostic models and treatment stratification frameworks; third, comprehensively investigating the crosstalk between Kcr and other post-translational modifications, and the regulatory influence of Kcr on the tumor–immune microenvironment interactions. Addressing these critical challenges offers significant potential to establish Kcr as a novel, actionable target in precision oncology, while facilitating the development of novel therapeutic strategies aimed at enhancing clinical outcomes in patients with cancer.
